# Relation of multi-marker panel to incident chronic kidney disease and rapid kidney function decline in African Americans: the Jackson Heart Study

**DOI:** 10.1186/s12882-018-1026-y

**Published:** 2018-09-20

**Authors:** Stanford E. Mwasongwe, Bessie Young, Aurelian Bidulescu, Mario Sims, Adolfo Correa, Solomon K. Musani

**Affiliations:** 1 Jackson Heart Study, Jackson State University, 350 W. Woodrow Wilson Ave., Suite 701, Jackson, MS 39213 USA; 2 Division of Nephrology, Kidney Research Institute University of Washington, Seattle, WA USA; 3 Veterans Affairs Puget Sound Health Care System, Seattle, WA USA; 4 Department of Medicine, University of Mississippi Medical Center, Jackson, MS USA; 5 Department of Epidemiology and Biostatistics, School of Public Health, Indiana University, Bloomington, IN USA

**Keywords:** Biomarker, Chronic kidney disease, Rapid kidney function decline, Estimated glomerular filtration rate, African Americans

## Abstract

**Background:**

Few investigations have evaluated the incremental usefulness of multiple biomarkers representing varying physiological pathways for predicting risk of renal outcomes in African Americans.

**Design, setting, participants, and measurements:**

We related a multi-marker panel to incident chronic kidney disease (CKD) and rapid kidney function decline (RKFD) in 2813 Jackson Heart Study participants without prevalent CKD at exam 1 (2000–2004) and with complete assays at exam 1 for 9 biomarkers: adiponectin, aldosterone, B-natriuretic peptide [BNP], cortisol, high sensitivity C-reactive protein (hsCRP), endothelin, homocysteine, plasma renin activity and mass. Incident CKD was defined as estimated glomerular filtration rate (eGFR) < 60 mL/min/1.73 m^2^ at exam 3 while RKFD was defined as eGFR ≥30% loss between exams 1 and 3 (8.2 median years). We employed multiple logistic regression model to describe association between the panel and incident CKD and RKFD and used backward elimination strategy to estimate the most parsimonious biomarker model while controlling for conventional risk factors.

**Results:**

The multi-marker panel predicted the risk for both incident CKD (odds ratios [OR], 2.72; 95% confidence intervals [CI], 1.63, 4.56; *P* = 0.001) and RKFD (2.61; 95% CI, 1.67, 4.08; *P <* 0.001). Per standard deviation increase in log biomarker concentrations were significantly (multivariable adjusted odds ratios, [95% confidence interval], *p*-value) associated with incident CKD: plasma adiponectin (1.24 [1.07, 1.44], *p* = 0.005) and leptin (1.3 [1.06, 1.61], *p* = 0.011), and with RKFD: plasma adiponectin (1.22 [1.06, 1.40], *p* = 0.006); hsCRP (1.17 [1.01, 1.36], *p* = 0.031) and aldosterone (0.85 [0.74, 0.96], *p* = 0.012). Moderate levels (3rd quartile) of aldosterone were inversely associated with incident CKD (0.54 [0.35, 0.82], *p* = 0.004) while leptin was associated with RKFD (1.64 [1.10, 2.44], *p* = 0.015). Biomarkers improved CKD risk prediction (*P* = 0.003) but not RKFD risk prediction (*P* = 0.10).

**Conclusion:**

In this community-based sample of African Americans, a multi-marker panel added only moderate predictive improvement compared to conventional risk factors.

**Electronic supplementary material:**

The online version of this article (10.1186/s12882-018-1026-y) contains supplementary material, which is available to authorized users.

## Background

Chronic kidney disease (CKD) is a significant health problem which is associated with increased morbidity and mortality, making its prevention a public health priority. Thirteen percent of the adult population of the United States have reduced kidney function or albuminuria [1]. Early identification of persons at greater risk of developing CKD is critical in prevention and management strategies. Traditionally, hypertension and diabetes are the most commonly known key risk factors for CKD. Others include advanced age, low high-density lipoprotein cholesterol (HDL) and metabolic syndrome [2]. Previous studies have shown that traditional factors alone are inadequate to explain CKD risks and improve risk stratification for CKD or progression of CKD [3–5]. Established CKD risk factors explain only 34% of renal disease progression among whites and 44% for African Americans after adjusting for sociodemographic, lifestyle and clinical factors [3, 6]. In clinical settings, CKD prognosis largely depends on traditional markers such as estimated glomerular filtration rate (eGFR) and albuminuria, however these biomarkers only offer modest risk prediction particularly in people with preserved levels of renal function [7] and are subject to intra-individual variability over time when hydration and medication use are involved. Additionally, albuminuria and eGFR can have a variable relationship, an example being the development of CKD (eGFR < 60 mL/min/1.73 m^2^) without albuminuria [8, 9]. Several other pathways may be involved in CKD development including inflammation and endothelial function [3, 10]. Studies in cardiovascular disease (CVD) and metabolic syndrome, have benefited from the use of circulating biomarkers in risk prediction [11–13]. Unlike biomarkers in CVD, the list of prognostic biomarkers in CKD is in continuous growth and the concept of a multi-marker approach has been proposed as single biomarkers are unable to fully describe changes in renal function [14]. While multi-marker approach to predict CKD has been reported in whites [10], the predictive value of models incorporating multiple biomarkers in CKD prediction among African Americans is not well studied.

In a community-based sample of African Americans enrolled in the Jackson Heart Study (JHS), we sought to identify biomarkers of interest and evaluate their incremental predictive value from a multi-marker panel representing physiological pathways implicated in kidney diseases: adiposity (adiponectin and leptin); adrenal (aldosterone and cortisol); endothelial function (endothelin and homocysteine); inflammation (C - reactive protein, [CRP]); natriuretic (B-type Natriuretic Peptide [BNP]) and renin angiotensin (plasma renin activity, and renin mass). We conducted tests on model improvement using both the C-statistic and the newer measures of net reclassification index (NRI) and integrated discrimination index (IDI) [15, 16].

## Methods

### Study sample

The JHS is a single-site community-based prospective study designed to identify risk factors for cardiovascular disease in African Americans. The recruitment details have been summarized previously [17, 18]. Briefly, the study enrolled 5306 participants ≥21 years of age (clinic Exam 1, September 2000 to March 2004) from urban and rural areas of three counties (Hinds, Madison, and Rankin) that comprises a Jackson, Mississippi Metropolitan Statistical Area (MSA). Participants were asked to return for a second clinic Exam (October 2005 to December 2008) and third clinic Exam (February 2009 to January 2013). The 9 biomarkers studied in this analysis were measured at Exam 1, while serum creatinine was measured at Exam 1 and 3 (8.2 median follow-up years). Analysis was restricted to 2813 participants after we excluded participants who (i) were missing serum creatinine values measured at Exam 1 and clinic Exam 3, *n* = 1548; (ii) had prevalent CKD at baseline or reported being on dialysis, *n* = 202; and (iii) were missing biomarker data, *n* = 743. Data were imputed for 206 participants with missing covariates. The institutional review board at the University of Mississippi Medical Center, Jackson State University, and Tougaloo College approved the study. All participants provided informed written consent.

### Definition of renal outcomes, biomarker selection and measurement

Incident CKD and RKFD, were both defined based on serum creatinine measured at Exams 1 and 3, as serum creatinine was not measured at the Exam 2. Serum creatinine was measured Exam 1 (2000–2004) and Exam 3 (2009–2013), serum creatinine was measured using a multipoint enzymatic spectrophotometric assay with the Vitros Ortho-Clinical Diagnostics Analyzer (Raritan, NJ). As part of the calibration study, measurement of serum creatinine were repeated for a random sample of 206 in 2006 using the enzymatic method on the Roche Modular P Chemistry Analyzer (Roche Diagnostics Corporation, Indianapolis, IN [19, 20]. Serum creatinine measured at Exam 1 was then calibrated to harmonize with serum creatinine measured at Exam 3 using isotope-dilution mass spectrometry traceable method [21]. We defined both endpoints based on the change between clinic Exams 1 and 3, and eGFR was estimated using Chronic Kidney Disease Epidemiology Collaboration (CKD-EPI) creatinine equation [21–23]. Incident CKD was defined as eGFR ≥60 mL/min/1.73 m^2^ at Exam 1 (i.e., no CKD at baseline) and eGFR < 60 mL/min/1.73 m^2^ at Exam 3 [24]. For RKFD, the difference expressed as a percentage of baseline eGFR represented progression if greater than 30% [2, 25]. Any eGFR > 120 mL/min/1.73 m^2^ was truncated to 120 mL/min/1.73 m^2^ to avoid large changes in those with high normal eGFRs [26, 27]. Positive values indicate a decline of eGFR from Exam 1 to Exam 3.

Nine biomarkers were selected because of the reported associations with kidney function, biologic plausibility and availability at first examination cycle. We measured high-sensitivity C-reactive protein (a marker of inflammation); adiponectin and leptin (adiposity); aldosterone, plasma renin activity, active renin mass concentration and B-type natriuretic peptide (markers of neuro-hormonal activity); homocysteine and endothelin (markers of endothelial function and oxidant stress). A detailed description of the standard assays were used for all biomarkers with coefficient of variations reported by Musani et al. [12]. Briefly, venous blood samples were withdrawn from study participants following 8-h fasting and stored at the JHS central repository in Minneapolis, MN, USA at − 70 °C until assayed [19, 28].

### Covariate assessments

The baseline examination included a complete medical history, physical examination and blood/urine collections. Prevalent diabetes was defined according to the American Diabetes Association (ADA) criteria as fasting (≥8 h) glucose ≥126 mg/dL, or hemoglobin A1c (HbA1C) ≥ 6.5% or use of diabetic medication (actual or self-reported) within 2 weeks prior to the clinic visit). Body mass index (BMI) was defined as the weight in kilograms divided by the height in meters squared. Clinic blood pressure (BP) was measured using random zero sphygmomanometer (Hawksley and Sons Ltd., Lancing, UK) following appropriate procedure; whereby the participants’ rested for 5 min in an upright position with their back and arms supported and a trained staff took two BP measurements in the right arm. The two clinic-measured BP were averaged to obtain the BP value used in the analysis. Fasting total and high-density lipoprotein (HDL) cholesterol were measured and quantified by an oxidase method and expressed as total to HDL ratio. Nephalometric immunoassay and enzymatic methods were used to quantify urinary albumin from a timed 24-h urine collection and a random spot morning urine collection [19]. Albuminuria was defined as a urinary-albumin-to-urinary-creatinine ratio (ACR) ≥30 mg/g using both methods. Current smoking status was defined as yes in participants who had smoked over 400 cigarettes in their lifetime and were actively smoking at the time of the baseline examination.

### Statistical analysis

All biomarkers were naturally log-transformed and standardized (mean = 0 and variance = 1) within sex to normalize their skewed distributions and also to account for sex-related differences. We performed separate analyses for incident CKD and RKFD by fitting 2 different models: i) the traditional model that consisted of known independent risk factors for CKD such as systolic blood pressure, hypertension, use of antihypertensive medication, current smoking status, body mass index, total cholesterol to HDL ratio, and diabetes; and ii) the biomarker model that consisted of CKD traditional risk factors and biomarkers. For the biomarker model, we first tested the relation of the entire biomarker panel with each outcome, and if the biomarker panel was statistically significant, we used backward elimination to identify a parsimonious subset of biomarkers that remained significantly associated with incident CKD and RKFD. The retained biomarkers were thereafter used to construct weighted multi-marker score following the approach applied by Wang et al. [29]. The sum of sex-standardized log-biomarker concentration weighed by the estimated regression coefficients of each selected biomarker constituted the risk score on a continuous scale. We then used the risk score as a continuous predictor or categorized using quartiles to evaluate its association with each outcome. For secondary analyses, we stratified by obesity status in our effort to understand obesity’s moderating effects on the association of biomarkers with the outcomes, considering that the Jackson Heart Study participants are on average obese (BMI > 30 kg/m^2^). Additionally, we compared the biomarkers distributions between included versus excluded participants using Wilcoxon rank-sum test.

To understand the utility of the biomarkers in the prediction of incident CKD and RKFD we compared the performance of the biomarker model with the traditional model. We computed performance metrics that included change in the C-statistic to assess model discrimination, and Integrated discrimination index (IDI) and net reclassification index (NRI) [16, 30] to assess reclassification improvement. IDI and NRI quantifies the model’s ability to predict outcome when biomarkers are included in addition to traditional risk factors. Calibration was evaluated with Hosmer-Lemeshow goodness-of-fit test with *P*-value < 0.05 indicating a poorly calibrated model. Data were imputed for participants with missing covariates (*n* = 206), with 20 data sets using fully conditional specification (FCS) [31]. In FCS, imputations are generated sequentially by specifying an imputation model for each variable given the other variables. In this way, FCS is suited for imputing data of variables with different scales and complex relations with each other. The percentage of missing for each variable included in the analysis was 0.07% for BMI, 0.11% for SBP, 5.65% for total cholesterol to HDL ratio, 0.78% for antihypertensive medication, 0.04% for diabetes and 0.78% for current cigarette smoking status. We used the SAS EG statistical software version 7.1 for all statistical tests and SAS macro developed by Kennedy and Pencina for model performance evaluation [32]. All statistical significance was defined as two-tailed *P* ≤ 0.05. For analysis of the structure of association between biomarkers and outcome, we used Generalized Additive Models implemented in R to check for non-linearity.

## Results

### Baseline study characteristics

**Fig. 1:**
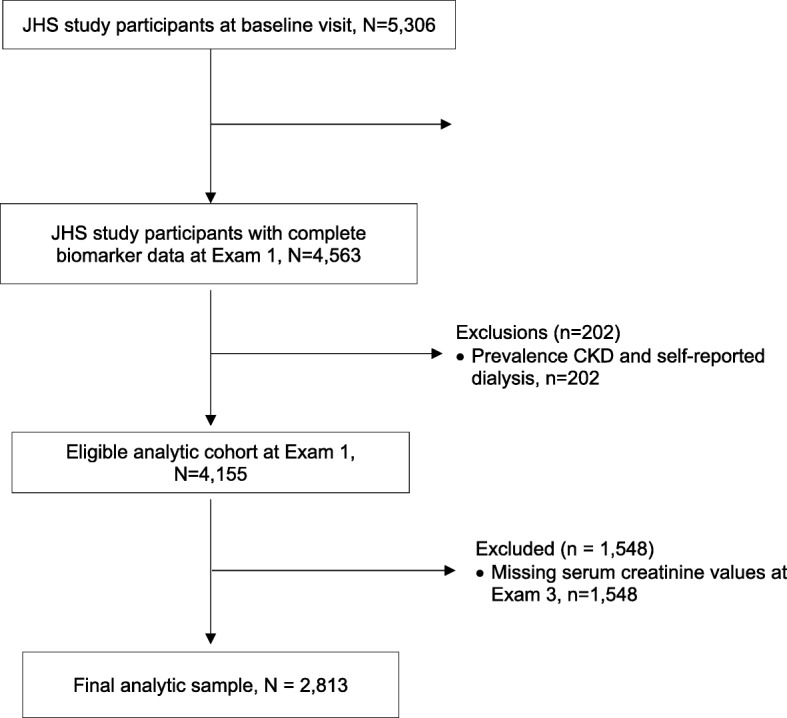
CONSORT flow diagram for the relation of multi-marker panel and incident chronic kidney disease (CKD) and rapid kidney function decline (RKFD)

Demographic, clinical and biomarker distributions at exam 1 are presented in Table 1**.** We included 2813 JHS participants in our analytic cohort based on various inclusion criteria. A consort diagram of the exclusions performed in our analyses is shown in Fig. 1. Mean age of the study cohort at baseline was 54 years, 62.8% were women, the mean body mass index (BMI) was 31.9 ± 7.3, while 16.6% had diabetes and 47.5% reported taking blood pressure medications. The average estimated GFR at baseline was 98.08 ± 17.76 ml/min/1.73m^2^. eGFR declined 9.93% (median 8.10; ranged from 0.00 to 18.00%) between Exam 1 and Exam 3 (8.2 median follow-up years).

**Table 1:** Baseline characteristics of study participants (*N* = 2607)

Characteristics	Mean ± SD
Age, years	53 ± 12
Female, % (n)	63 (1636)
BMI, kg/m^2^	31.9 ± 7.3
SBP, mmHg	125.9 ± 15.7
Baseline eGFR, mL/min per 1.73 m^2^	98.1 ± 17.7
Total cholesterol to HDL ratio	4.1 ± 1.3
Blood pressure medications, % (n)	47.5 (1238)
Diabetes, % (n)	16.6 (432)
Current smoking, % (n)	11.7 (306)
Biomarker level	Median (25th, 75th percentiles)
Adiponectin, ng/mL	4037.0 (2640.1, 6339.2)
Aldosterone, ng/mL	4.3 (2.5, 6.9)
BNP, pg/mL	6.7 (2.3, 14.8)
hsCRP, mg/dL	0.3 (0.1, 0.6)
Endothelin, pg/mL	1.2 (0.9, 1.6)
Homocysteine, μmol/dL	8.4 (7.2, 9.9)
Leptin, ng/mL	22.9 (10.1, 39.3)
Plasma renin activity, ng/mL/hr	0.4 (0.2, 1.0)
Active renin mass concentration, pg/mL	6.7 (5.1, 9.4)

Data presented as mean ± standard deviation (SD) for continuous variables and percentage (count) for dichotomous variables unless otherwise indicated

*Abbreviations*: *BMI* body mass index, *SBP* systolic blood pressure, *eGFR* estimated glomerular filtration rate, *HDL* high-density lipoprotein cholesterol, *hsCRP* high-sensitive c-reactive protein, *BNP* B-type natriuretic peptide

#### Association of multi-marker panel with incident CKD

**Fig. 2:**
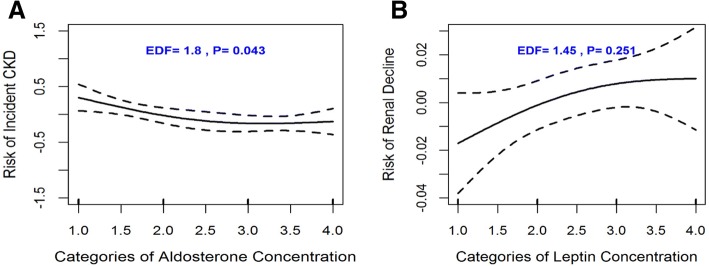
Penalized spline smoother of the relationship between the risk of incident chronic kidney disease (CKD) and aldosterone (**a**), and between RKFD and leptin (**b**)

During a median follow-up period of 8.2 years, 10.5% (*n* = 178 women) of participants developed incident CKD. We observed that the multi-marker panel was significantly associated with the development of CKD (*P =* 0.004) on follow-up. Upon backward elimination, continuous log plasma adiponectin (*P* = 0.005) and leptin concentrations (*P* = 0.011) were retained as significantly associated with incident CKD. We also tested the association of high levels (based on data derived quartiles) of plasma adiponectin and leptin with incident CKD. The multivariable adjusted odds ratios (ORs) and 95% confidence intervals (95% CI) are summarized in Table 2. High levels of log plasma adiponectin (OR, 1.66; 95% CI, 1.08–2.55) and leptin (OR, 2.00; 95% CI 2.00; *P*-value = 0.022); 95% CI, 1.18–3.38; *P*-value = 0.009) were significantly associated with incident CKD. In addition, moderate levels (second quartile) of plasma adiponectin were also significantly associated with incident CKD. When we combined adiponectin and leptin to form a multi-marker score, and found that the ORs almost doubled for the continuous multi-marker score. Both high and moderate levels of the multi-marker score were significantly associated with incident CKD. In Fig. 2a, the smoother splines show the structure of the multivariable relationship between plasma aldosterone with CKD. Moderate level of plasma aldosterone appeared to be protective against incident CKD development.

**Table 2:** Associations of multi-marker panel, individuals’ biomarkers and multi-marker scores with incident CKD and rapid kidney function decline

Biomarkers	Incident CKD			Rapid Kidney Function Decline (RKFD)		
	Cases/ # at risk	Multivariable Adjusted Odds Ratio (95% CI)	*P*-value	Cases/ # at risk	Multivariable Adjusted Odds Ratio (95% CI)	*P*-value
Entire panel		2.72 (1.63, 4.56)	0.001		2.61 (1.67, 4.08)	0.001
Adiponectin						
*Continuous*		1.24 (1.07, 1.44)	0.005		1.22 (1.06, 1.40)	0.006
Q1	54/703	Reference		63/703	Reference	
Q2	71/703	1.37 (0.89, 2.09)	0.151	78/703	1.20 (0.83, 1.75)	0.330
Q3	72/704	1.31 (0.85, 2.02)	0.217	72/704	1.14 (0.77, 1.67)	0.515
Q4	98/703	1.66 (1.08, 2.55)	0.022	97/703	1.49 (1.02, 2.18)	0.045
Leptin						
*Continuous*		1.31 (1.06, 1.61)	0.011		1.12 (0.93, 1.34)	0.234
Q1	54/698	Reference	…	67/698	Reference	…
Q2	79/711	1.37 (0.89, 2.11)	0.151	77/711	1.13 (0.77, 1.66)	0.525
Q3	73/699	1.52 (0.95, 2.42)	0.079	90/699	1.64 (1.10, 2.44)	0.015
Q4	89/705	2.00 (1.18, 3.38)	0.009	76/705	1.29 (0.80, 2.06)	0.295
hsCRP						
*Continuous*		1.13 (0.96, 1.33)	0.149		1.17 (1.01, 1.36)	0.031
Q1	53/700	Reference	…	56/700	Reference	…
Q2	88/707	1.22 (0.80, 1.87)	0.358	81/707	1.12 (0.76, 1.65)	0.572
Q3	85/705	1.32 (0.86, 2.02)	0.212	88/705	1.25 (0.85, 1.84)	0.258
Q4	69/701	1.13 (0.71, 1.80)	0.593	85/701	1.28 (0.85, 1.92)	0.230
Aldosterone						
*Continuous*		0.92 (0.8,1.06)	0.229		0.85 (0.74, 0.96)	0.012
Q1	77/698	Reference	…	93/698	Reference	…
Q2	68/720	0.82 (0.55, 1.23)	0.347	80/720	0.88 (0.62,1.24)	0.461
Q3	58/707	0.54 (0.35, 0.82)	0.004	66/707	0.66 (0.46, 0.95)	0.026
Q4	92/688	0.68 (0.46, 1.04)	0.077	71/688	0.62 (0.43, 0.89)	0.009
Multi-marker Score						
0	40/651	Reference		42/651	Reference	
1	53/652	1.48 (0.94–2.33)	0.093	54/652	1.12 (0.75–1.67)	0.586
2	73/652	2.02 (1.30–3.14)	0.002	74/652	1.62 (1.11–2.37)	0.001
3	90/652	2.45 (1.53–3.91)	<.001	95/652	2.04 (1.40–2.99)	<.001

*Abbreviations*: *Q1* quartile 1, *Q2* quartile 2, *Q3* quartile 3, *Q4* quartile 4, *hsCRP* high-sensitive C-reactive protein

Incident chronic kidney disease (CKD) was defined a decline from eGFR ≥60 mL/min/1.73 m^2^ at exam1 to eGFR < 60 mL/min/1.73 m^2^ at exam 3 follow-up (median follow-up duration: 8.0 years)

Rapid kidney function decline (RKFD) was defined as a decline in estimated glomerular filtration rate (eGFR) ≥ 30% from exam 1 to exam 3 (median follow-up duration: 8.0 years)

Multivariate model for the estimation of ORs were for adjusted for age, sex, baseline estimated glomerular filtration rate (eGFR), systolic blood pressure, hypertension, use of hypertension medication, smoking, body mass index (BMI), total cholesterol to high-density lipoprotein cholesterol (HDL) ratio and diabetes

#### Association of multi-marker panel with rapid kidney function decline

During a median follow-up period of 8.2 years, 11.0% (*n* = 202 women) of participants developed RKFD, and the multi-marker panel was significantly associated with RKFD (*p*-value = 0.001). Adiponectin and aldosterone were retained as significant correlates of RKFD (Table 2). When divided into quartiles, with exception of aldosterone, which was protective (OR, 0.62; 95% CI, 0.43–0.89; *P*-value = 0.009), the highest quartile for adiponectin (OR, 1.49; 95% CI, 1.10–2.44; *P*-value =0.045) and medium quartile leptin level (OR, 1.64; 95% CI, 1.10–2.44; *P*-value = 0.015) were not significantly associated with RKFD. A multi-marker score that comprise the three biomarkers was significantly associated with the risk for RKFD both as a continuous and categorical states. A smoother splines, Fig. 2b depict the multivariable relationship between leptin with RKFD.

### Secondary analyses

For secondary analyses, we compared the biomarkers distributions of participants included versus those excluded from the analyses due and results are summarized on Additional file 1: Table S1. We also repeated the association analyses stratified by obesity status to assess whether obesity moderates the biomarker-incident CKD / RKFD relation. Only adiponectin concentrations interacted significantly with obesity status (*P*-value = 0.016). Results of the analysis stratified by obesity status, showed that high levels of both adiponectin and medium level of leptin were significantly associated with development of CKD among non-obese participants but not among obese participants. Similar results were evident for the multi-marker score combining adiponectin and leptin although among obese participants, the highest quartile (4th) of the risk score was also significantly associated with CKD. Stratified analyses of the association of adiponectin, aldosterone and leptin with RKFD showed a similar pattern as for incident CKD. In the leptin-RKFD relation however, the second quartile was significantly associated with RKFD but not the other quartiles suggesting possible non-linear relation (Additional file 1: Table S3).

#### Evaluation of model performance

The added predictive ability of the biomarker model in terms of improved discrimination and reclassification above the conventional CKD risk factors is shown in Table 3**.** The biomarker model exceeds the traditional CKD risk factor model by 1% in predicting incident CKD and RKFD. With respect to reclassification improvement, the biomarker model reclassified 11% and 15% CKD and RKFD events, respectively compared to the traditional model. Moreover, the predicted mean probability of events was significantly different between biomarker and traditional CKD risk factor models, with the former performing better than the later.

**Table 3:** Incremental predictive utility of biomarkers for incident chronic kidney disease (CKD), rapid kidney function decline (RKFD) showing C-statistics and reclassification metrics

	C-Statistics	NRI		IDI		Calibration Statistics^a^ (χ^2^, *P*)
		Events *correctly reclassified*	Non-Events *correctly reclassified*	Mean *probability* for events	Mean *probability* for non-events	
Chronic kidney disease (CKD)						
Model 1: age-sex-MV^b^	0.87	11%	5%	32%	7.4%	12.93 (*P* = 0.11)
Model 2: age-sex-MV-Biomarker^c^	0.88			33%	7.3%	14.26 *(P* = 0.08)
*P*-value comparing models 1 vs. 2	0.003	0.08	0.01	0.01		
Rapid kidney function decline						
Model 1: age-sex-MV	0.76	15%	11%	19.3%	9%	12.19 (*P* = 0.14)
Model 2: age-sex-MV-Biomarker^d^	0.77			20.3%	9%	18.42 (*P* = 0.02)
*P*-value comparing models 1 vs. 2	0.10	0.01	<.0001	0.0001		

*Abbreviations*: *NRI* net reclassification index, *IDI* integrated discrimination index

^a^A Hosmer-Lemeshow goodness-of-fit test indicate poor calibration if *P*-value < 0.05

^b^MV adjusted for age, sex, baseline estimated glomerular filtration rate (eGFR), systolic blood pressure, hypertension, use of hypertension medication, smoking, body mass index (BMI), total cholesterol to high-density lipoprotein cholesterol (HDL) ratio and diabetes

^c^In backward elimination of the biomarker panel, adiponectin and leptin are significant

^d^In backward elimination of the biomarker panel, adiponectin, high-sensitive C - reactive protein (CRP) and aldosterone are significant

## Discussion

We investigated the relation of a multi-marker panel with the development of incident CKD and RKFD in a large community-based sample of African Americans. We observed that a panel consisting of nine circulating biomarkers (adiponectin, aldosterone, BNP, hsCRP, endothelin, homocysteine, leptin, PRA, ARM) representing several distinct biologic pathways was associated with development of CKD and RKFD. We identified a smaller subset of biomarkers representing adiposity (adiponectin, leptin); and RAS (aldosterone) pathways that were also associated with these outcomes. Plasma adiponectin and leptin were both associated with development of CKD while plasma aldosterone had a protective effects against both CKD development and RKFD. The addition of biomarkers only marginally improved model discrimination and reclassification compared to the model with traditional risk factors as demonstrated by the small change in C-statistic and reclassification indices. In secondary analyses stratified by obesity status, selected biomarkers were significantly associated with incident CKD and RKFD in non-obese participants only, suggesting modification by obesity status.

In our study, adiponectin and leptin, two of the key cytokines secreted by adipocytes, predicted the development of incident CKD in a multivariable adjusted model. These findings are consistent with previous studies that investigated their association with CKD. In a case-control study among Chinese and Indian adults, patients with CKD had higher levels of leptin and adiponectin compared to controls [33]. Similar findings were also found in a patient population comprised of 60% African Americans in the greater New Orleans, Louisiana region, after adjusting for race and other risk factors associated with kidney disease [34]. To the contrary, other studies have also reported no difference in adiponectin levels [35–37]. The link between adipokines and changes in glomerular filtration rate has been reported previously [38]. Through endothelial dysfunction, oxidative stress and changes in immune response and inflammation, the adipokines are involved in kidney damage [39]. While serum aldosterone was reported to have weak but significant association with lower eGFR in Framingham Offspring Study, an inverse association where aldosterone appear to be protective was observed in this present study. Aldosterone’s conflicting results are also reported in the Ohasama Study where the authors attributed the lack of association of aldosterone with eGFR to a high salt-intake resulting from high sodium dietary conditions [40, 41].

Studies on single biomarkers have reported on the relation of CRP and aldosterone with kidney function. Works by Fox and colleagues as well as Shankar and others showed that CRP is associated with prevalent CKD but not with the development of CKD [42, 43]. While previous studies both clinical and observational have demonstrated CRP’s pathogenic role in renal damage [44, 45], in the current analysis, CRP was not associated with the development of either CKD or RKFD. Hannemann and colleagues found an inverse association of plasma aldosterone concentration with eGFR in the general population [46]. In the present study, participants with medium level quartile and higher aldosterone level had 46% (*P =* 0.004) and 38% (*P*-value = 0.009) less likely to develop CKD or experience RKFD, respectively. When stratified by obesity status, biomarkers were associated with development of CKD and RKFD in non-obese, particularly for leptin and adiponectin. Biomarkers linkages to the development of CKD in the absence of obesity has been reported before even though the mechanism is poorly understood [38, 47].

Few community-based studies have evaluated kidney disease biomarkers to assess their usefulness in stratifying disease risk [3]. We undertook this study to address this gap in CKD literature. Data from the Framingham Heart Study (FHS) followed a multi-marker approach to predict incident CKD and microalbuminuria. A panel of seven biomarkers (C-reactive protein, aldosterone, renin, BNP, plasminogen-activator inhibitor type 1, fibrinogen, and homocysteine) was associated with the development of CKD with homocysteine and aldosterone retained as significant markers in the backward elimination model [10]. Our data extends these findings to a large community-based sample of African Americans in Mississippi. Unlike FHS where homocysteine and aldosterone were retained as significant markers for incident CKD prediction, in JHS adiponectin and leptin were the significant markers.

When comparing indices of model improvement the biomarker model was associated with the same change in C-statistic (ΔC = 0.01) for prediction of incident CKD and RKFD. Researchers generally consider a change in C-statistic of at least 0.05 as indicative of a predictor with clinical significance [48]. While C statistics has been criticized for being insensitive to small changes in predictive accuracy [49], it was preferred here to permit easy comparison with findings in the literature, which often used the metric [50]. We also computed the NRI and IDI indices to complement C-statistic. Consistent with results based on C-statistics for the biomarker model, relative IDI had small but significant incremental predictive ability that was also higher than that reported in FHS. NRI though statistically non-significant was higher than that reported in FHS (JHS NRI = 16.1%, *P* = 0.08; FHS NRI = 6.9%, *P* = 0.0004). Though the metrics of model improvement are study/cohort specific, suffice it to say that they hold promise for CKD prediction in African Americans as is in white populations. The utility of biomarkers in improving disease prediction is highly successful in the area of cardiovascular medicine [50–52]; however, the yield has been relatively small in women and the elderly [53, 54]. With exception of Velagaleti et al. report on prediction of heart failure (ΔC =0.02) [55], most CVD research has reported lower incremental benefit compared to current analyses. This may be because CVD risk factors are well characterized and the existence of multiple risk-algorithms aid prediction, something which is lacking in CKD research.

### Strengths and limitations

This study has some strengths and limitations. The analyses had a large sample size and a well-documented spectrum of biomarkers. We also adjusted for many CKD factors so the independent association between multi-marker panel and CKD development could be assessed. Some limitations require mentioning. Our sample was primarily African Americans, limiting generalizability to other ethnicities. JHS was designed to investigate CVD risk factors, thus the biomarkers collected were not specifically for CKD, although CVD is a potent risk factor for CKD and CKD progression. Finally, with the study being observational in nature, it is possible that the CKD being detected might have developed at an earlier date.

## Conclusions

In summary, while the predictive utility of biomarkers in these data were minimal, they do not exclude the role of using circulating biomarkers to provide insight into early development of CKD in this vulnerable population. Circulating adipokines (adiponectin, leptin), CRP and aldosterone biomarkers incrementally predicted incident CKD and RKFD in our large community-based sample.

## Additional file

Additional file 1: **Table S1.** Comparing characteristics of the Included versus Excluded Participants. **Table S2.** Baseline characteristics and biomarkers distribution by incident chronic kidney disease (CKD) and rapid kidney function decline (RKFD). **Table S3.** Associations between biomarkers with incident CKD and RKFD stratified by obesity status. (DOCX 39 kb)

## Abbreviations

ACR: Albumin-creatinine ratio
ADA: American Diabetes Association
BMI: Body mass index
BNP: B-natriuretic peptide
BP: Blood pressure
CKD: Chronic kidney disease
CKD-EPI: Chronic Kidney Disease Epidemiology Collaboration creatinine equation
CVD: Cardiovascular disease
eGFR: Estimated glomerular filtration rate
FCS: Fully conditional specification
HDL: High-density lipoprotein
hsCRP: High sensitive C-reactive protein
IDI: Integrated discrimination index
JHS: Jackson Heart Study
NRI: Net reclassification index
RKFD: Rapid kidney function decline
