# Supplementary material for: Relation of multi-marker panel to incident chronic kidney disease and rapid kidney function decline in African Americans: the Jackson Heart Study
Source: BMC Nephrol. 2018 Sep 20;19:239. doi: 10.1186/s12882-018-1026-y (PMC6147037; doi:10.1186/s12882-018-1026-y)
Supplement: Supplementary file 1 — Table S1. Comparing characteristics of the Included versus Excluded Participants. Table S2. Baseline characteristics and biomarkers distribution by incident chronic kidney disease (CKD) and rapid kidney function decline (RKFD). Table S3. Associations between biomarkers with incident CKD and RKFD stratified by obesity status. (DOCX 39 kb) [file 12882_2018_1026_MOESM1_ESM.docx]

**Additional file 1**

**Relation of multi-marker panel to the development of incident chronic kidney disease and rapid kidney function decline in African Americans: the Jackson Heart Study**

**Table S1**. Comparing characteristics of the Included versus Excluded Participants

**Table S2:** Baseline characteristics and biomarkers distribution by incident chronic kidney disease (CKD) and rapid kidney function decline (RKFD)

**Table S1. Comparing characteristics of the Included and Excluded Participants**

| *Characteristics* | *Included Participants, Mean Score* | *Excluded Participants,*  *Mean Score* | *P-value* |
| --- | --- | --- | --- |
| Adiponectin, ng/mL | 2460.38 | 2667.74 | <0.001 |
| Aldosterone, ng/mL | 2251.82 | 2650.40 | 0.018 |
| BNP, pg/mL | 1959.78 | 2367.45 | <.001 |
| hsCRP, mg/mL | 2559.71 | 2649.64 | 0.031 |
| Endothelin, pg/dL | 2464.66 | 2760.58 | <.001 |
| Homocysteine, µmol/L | 2395.97 | 2817.06 | <.001 |
| Leptin, ng/mL | 2568.64 | 2604.53 | 0.389 |
| Plasma Renin Activity, ng/mL/hr | 1142.08 | 1240.35 | 0.001 |
| Active renin mass concentration, pg/mL/hr | 1360.05 | 1452.69 | 0.002 |
| **Abbreviations**: BNP, B-type natriuretic peptide; hsCRP, high sensitive C-reactive protein | | | |

| **Table S2. Baseline characteristics and biomarkers distributions by incident chronic disease and rapid kidney function decline status and participants with both CKD-RKFD** | | | | | | | |
| --- | --- | --- | --- | --- | --- | --- | --- |
| **Characteristics** | **CKD** | | | **RKFD** | | | **CKD-RKFD Overlap (n=185)** |
|  | **No** | **Yes** | ***P*-value** | **No** | **Yes** | ***P*-value** |  |
| Age, yr | 52.4 (51.9-52.9) | 63.9 (62.8, 65.1) | <.001 | 52.7 (52.3, 53.2) | 60.8 (59.6, 62.1) | <.001 | 63.7 (10.6) |
| Female, % | 33.7 (947) | 4.2 (117) | 0.492 | 33.9 (956) | 3.8 (108) | 0.251 | 61.6 (114) |
| BMI, Kg/m^2^ | 55.9 (1571) | 6.3 (178) | 0.492 | 31.8 (31.5, 32.1) | 32.3 (31.5, 33.1) | 0.282 | 32.2 (5.9) |
| Systolic BP, mmHg | 125 (124.4, 125.6) | 135 (133.1, 137) | <.001 | 125.2 (124.6, 125.8) | 132.8 (130.8, 134.7) | <.001 | 135.7 (18.1) |
| eGFR, ml/min per 1.73 m^2^ | 100.3 (99.6, 100.9) | 80.2 (78.4, 82.01) | <.001 | 98.5 (97.8, 99.2) | 95.4 (93.5, 97.4) | 0.004 | 86.8 (15.8) |
| Total Cholesterol to HDL ratio | 4.06 (4.01, 4.11) | 4.22 (4.07, 4.38) | 0.041 | 4.1 (4.0, 4.1) | 4.1 (3.9, 4.3) | 0.437 | 4.2 (1.3) |
| BP Medications, % | 40.9 (1150) | 7.5 (212) | <.001 | 40.7 (1,145) | 7.7 (217) | <.001 | 75.7 (140) |
| Diabetes, % | 14.8 (417) | 3.8 (108) | <.001 | 14.0 (395) | 4.6 (130) | <.001 | 43.2 (80) |
| Current smoking, % | 10.9 (306) | 1.03 (306) | 0.244 | 10.2 (288) | 1.7 (47) | 0.061 | 22 (11.9) |
| **Biomarkers level, median scores (25^th^, 75^th^ percentile scores** | | | | | | | |
| Adiponectin, ng/mL | 1385.7 | 1589.1 | <.001 | 1561.7 | 1387.8 | 0.004 | 4695.4 (3106.4, 7453.2) |
| Aldosterone, ng/mL | 1397.9 | 1484.5 | 0.083 | 1326.2 | 1417.0 | 0.063 | 4.1 (2.3, 7.4) |
| BNP, pg/mL | 1367.1 | 1748.02 | <.001 | 1609.5 | 1381.9 | <.001 | 11.5 (4.5, 26.9) |
| hsCRP, mg/mL | 1399.5 | 1471.2 | 0.152 | 1552.1 | 1389.0 | 0.001 | 0.32 (0.16, 0.56) |
| Endothelin, pg/dL | 1388.2 | 1567.5 | 0.003 | 1564.1 | 1387.5 | 0.003 | 1.30 (1.10, 1.70) |
| Homocysteine, µmol/L | 1353.3 | 1865.1 | <.001 | 1611.3 | 1381.7 | <.001 | 9.20 (8.10, 11.20) |
| Leptin, ng/mL | 1400.8 | 1459.9 | 0.237 | 1465.8 | 1399.7 | 0.177 | 23.90 (10.5, 40.0) |
| ^¥^Plasma Renin Activity, ng/mL/hr | 655.2 | 705.3 | 0.099 | 727.7 | 651.3 | 0.011 | 0.50 (0.20, 1.50) |
| ^¥^Active renin mass concentration, pg/mL/hr | 738.9 | 823.9 | 0.041 | 824.3 | 738.3 | 0.032 | 7.85 (5.30, 12.10) |
| Data presented as mean (SD) for continuous variables and percentage (n) for categorical variables. Biomarkers distributions are presented in median scores with additional 25^th^ and75th percentiles indicated for participants with both CKD-RKFD status.  ¥The number of overlapping participants for Renin Activity RIA and Renin Mass IRMA were 107 and 78 respectively.  **Abbreviations**: CKD, chronic kidney disease, RKFD, rapid kidney function decline; BMI, body mass index; BP, blood pressure; eGFR, estimated glomerular filtration rate; HDL, high density lipoprotein; BNP, B-type natriuretic peptide; hsCRP, high-sensitive c-reactive protein | | | | | | | |

**Table S3. Associations between biomarkers with incident CKD and RKFD stratified by obesity status**

| *Biomarkers* | *Multivariable Adjusted Odds Ratio (95% CI)* | *P-value* | *Multivariable Adjusted Odds Ratio (95% CI)* | *P-value* | *P-value for Interaction* |
| --- | --- | --- | --- | --- | --- |
|  | *Non-Obese Participants* | | *Obese Participants* | |  |
| Adiponectin | | | | | |
| Q1 | Reference |  | Reference |  | 0.016 |
| Q2 | 1.27 (0.61-2.65) | 0.520 | 1.43 (0.84-2.45) | 0.190 |  |
| Q3 | 1.23 (0.61, 2.51) | 0.560 | 1.38 (0.79-2.42) | 0.256 |  |
| Q4 | **2.30 (1.17-4.53)** | 0.016 | 1.24 (0.69-2.25) | 0.473 |  |
| Leptin | | | | | |
| Q1 | Reference |  | Reference |  | 0.507 |
| Q2 | 1.38 (0.81-2.35) | 0.237 | 1.11 (0.54, 2.29) | 0.786 |  |
| Q3 | **2.44 (1.28-4.65)** | 0.007 | 1.44 (0.72, 2.91) | 0.309 |  |
| Q4 | 1.54 (0.60-3.90) | 0.367 | 1.13 (0.55, 2.34) | 0.739 |  |
| CRP | | | | | |
| Q1 | Reference | … | Reference | … | 0.945 |
| Q2 | 0.84 (0.47, 1.50) | 0.556 | 1.94 (0.96, 3.91) | 0.064 |  |
| Q3 | 1.28 (0.71, 2.30) | 0.411 | 1.56 (0.77, 3.13) | 0.215 |  |
| Q4 | 0.84 (0.42, 1.68) | 0.622 | 1.56 (0.76, 3.19) | 0.223 |  |
| Aldosterone | | | | | |
| Q1 | Reference |  | Reference | … | 0.903 |
| Q2 | 0.79 (0.44, 1.45) | 0.449 | 0.93 (0.52, 1.65) | 0.805 |  |
| Q3 | 0.63 (0.34, 1.16**)** | 0.137 | **0.51 (0.28, 0.93)** | 0.027 |  |
| Q4 | 0.64 (0.35, 1.18) | 0.155 | 0.76 (0.43, 1.32) | 0.322 |  |
| Multi-marker Score |  |  |  |  |  |
| ***Continuous*** | 4.11 (1.81-9.29) | 0.001 | 2.11 (0.96-4.62) | 0.06 |  |
| Categorical Risk Score | | | | | |
| *Biomarkers* | *Multivariable Adjusted Odds Ratio (95% CI)* | *P-value* | *Multivariable Adjusted Odds Ratio (95% CI)* | *P-value* | *P-value for Interaction* |
| 0 | Reference | … | Reference | … |  |
| 1 | 1.00 (0.53-1.89) | 0.990 | 1.91 (0.79-4.59) | 0.150 |  |
| 2 | 1.75 (0.93-3.29) | 0.080 | 2.21 (0.95-5.12) | 0.065 |  |
| 3 | 3.19 (1.59-6.39) | 0.001 | 2.37 (1.01-5.58) | 0.048 |  |
| **Rapid kidney function decline** | | | | | |
|  | *Non-Obese Participants* | | *Obese Participants* | |  |
| Adiponectin | | | | | |
| Q1 | Reference | … | Reference | … | 0.049 |
| Q2 | 1.11 (0.56, 2.20) | 0.760 | 1.24 (0.78, 1.95) | 0.362 |  |
| Q3 | 1.32 (0.68, 2.54) | 0.409 | 1.06 (0.65, 1.73) | 0.821 |  |
| Q4 | **1.91 (1.02, 3.60)** | **0.044** | 1.15 (0.69, 1.92) | 0.597 |  |
| CRP |  |  |  |  |  |
| Q1 | Reference | … | Reference | … | 0.739 |
| Q2 | 0.93 (0.54, 1.61) | 0.798 | 1.47 (0.81, 2.67) | 0.209 |  |
| Q3 | 1.56 (0.92, 2.65) | 0.097 | 1.18 (0.65, 2.14) | 0.583 |  |
| Q4 | 1.16 (0.62, 2.17) | 0.634 | 1.43 (0.79, 2.59) | 0.241 |  |
| Aldosterone | | | | | |
| Q1 | Reference | … | Reference | … | 0.915 |
| Q2 | 0.93 (0.56-1.54) | 0.778 | 0.89 (0.55, 1.42) | 0.620 |  |
| Q3 | 0.71 (0.41-1.22) | 0.213 | 0.67 (0.41, 1.09) | 0.108 |  |
| Q4 | **0.49 (0.27-0.90)** | **0.022** | 0.71 (0.44, 1.14) | 0.157 |  |
| Leptin | | | | | |
| Q1 | Reference |  | Reference | … | 0.552 |
| Q2 | 1.27 (0.77, 2.10) | 0.341 | 1.11 (0.54, 2.29) | 0.786 |  |
| Q3 | **2.28 (1.26, 4.13)** | **0.006** | 1.44 (0.72, 2.91) | 0.304 |  |
| *Biomarkers* | *Multivariable Adjusted Odds Ratio (95% CI)* | *P-value* | *Multivariable Adjusted Odds Ratio (95% CI)* | *P-value* |  |
| Q4 | 1.35 (0.52, 3.53) | 0.542 | 1.13 (0.55, 2.34) | 0.739 |  |
| Multi-marker Score | | | | | |
| ***Continuous*** | 4.05 (1.99-8.23) | 0.001 | 1.92 (0.99-3.75) | 0.060 |  |
| Categorical Risk Score | | | | | |
|  | *Non-Obese Participants* | | *Obese Participants* | |  |
| 0 | Reference | … | Reference | … |  |
| 1 | 1.23 (0.63-2.41) | 0.540 | 1.21 (0.67-2.19) | 0.520 |  |
| 2 | **1.95 (1.01-3.75)** | **0.045** | 1.58 (0.91-2.76) | 0.110 |  |
| 3 | **3.00 (1.61-5.59)** | **0.001** | **1.95 (1.11-3.44)** | **0.021** |  |
